# Mechanochemical synthesis of air-stable hexagonal Li_4_SnS_4_-based solid electrolytes containing LiI and Li_3_PS_4_†

**DOI:** 10.1039/d1ra06466e

**Published:** 2021-12-03

**Authors:** Misae Otoyama, Kentaro Kuratani, Hironori Kobayashi

**Affiliations:** Department of Energy and Environment, Research Institute of Electrochemical Energy, National Institute of Advanced Industrial Science and Technology (AIST) 1-8-31, Midorigaoka, Ikeda Osaka 563-8577 Japan misae-otoyama@aist.go.jp +81-72-751-9609 +81-72-751-7932

## Abstract

Sulfide solid electrolytes with high ionic conductivity and high air stability must be developed for manufacturing sulfide all-solid-state batteries. Li_10_GeP_2_S_12_-type and argyrodite-type solid electrolytes exhibit a high ionic conductivity of ∼10^−2^ S cm^−1^ at room temperature, while emitting toxic H_2_S gas when exposed to air. We focused on hexagonal Li_4_SnS_4_ prepared by mechanochemical treatment because it comprises air-stable SnS_4_ tetrahedra and shows higher ionic conductivity than orthorhombic Li_4_SnS_4_ prepared by solid-phase synthesis. Herein, to enhance the ionic conductivity of hexagonal Li_4_SnS_4_, LiI was added to Li_4_SnS_4_ by mechanochemical treatment. The ionic conductivity of 0.43LiI·0.57Li_4_SnS_4_ increased by 3.6 times compared with that of Li_4_SnS_4_. XRD patterns of Li_4_SnS_4_ with LiI showed peak-shifting to lower angles, indicating that introduction of I^−^, which has a large ionic radius, expanded the Li conduction paths. Furthermore, Li_3_PS_4_, which is the most air-stable in the Li_2_S–P_2_S_5_ system and has higher ionic conductivity than Li_4_SnS_4_, was added to the LiI–Li_4_SnS_4_ system. We found that 0.37LiI·0.25Li_3_PS_4_·0.38Li_4_SnS_4_ sintered at 200 °C showed the highest ionic conductivity of 5.5 × 10^−4^ S cm^−1^ at 30 °C in the hexagonal Li_4_SnS_4_-based solid electrolytes. The rate performance of an all-solid-state battery using 0.37LiI·0.25Li_3_PS_4_·0.38Li_4_SnS_4_ heated at 200 °C was higher than those obtained using Li_4_SnS_4_ and 0.43LiI·0.57Li_4_SnS_4_. In addition, it exhibited similar air stability to Li_4_SnS_4_ by formation of LiI·3H_2_O in air. Therefore, addition of LiI and Li_3_PS_4_ to hexagonal Li_4_SnS_4_ by mechanochemical treatment is an effective way to enhance ionic conductivity without decreasing the air stability of Li_4_SnS_4_.

## Abstract 2

Addition of LiI and Li_3_PS_4_ to hexagonal Li_4_SnS_4_ enhances ionic conductivity without decreasing the air stability of Li_4_SnS_4_.

## Graphical abstract

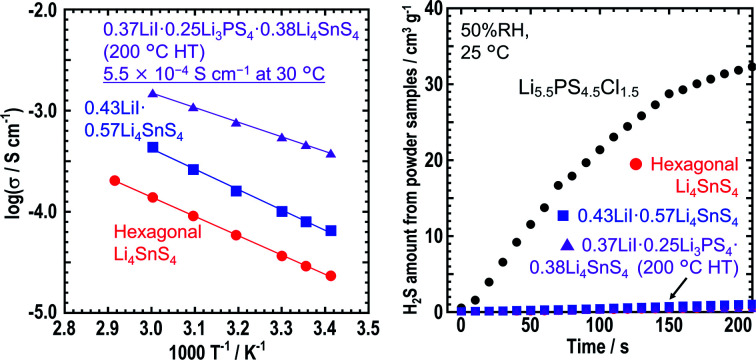

## Introduction

High performance all-solid-state lithium batteries are desirable to realize safe and large-scale power sources of electric vehicles and energy storage systems.^1,2^ Compared with oxide solid electrolytes, sulfide solid electrolytes have attracted significant attention because they show higher ionic conductivities, especially in LGPS-type (*e.g.* Li_10_GeP_2_S_12_ (12 mS cm^−1^)^3^ and Li_9.54_Si_1.74_P_1.44_S_11.7_Cl_0.3_ (25 mS cm^−1^)^4^), argyrodite-type (*e.g.* Li_5.5_PS_4.5_Cl_1.5_ (12 mS cm^−1^)^5^), and Li_2_S–P_2_S_5_ systems (*e.g.* Li_7_P_3_S_11_ (17 mS cm^−1^)^6,7^). Moreover, their high deformability enables the development of sufficient solid–solid contacts by cold pressing.^8,9^ However, the low air stability of sulfide solid electrolytes increases the risks of toxic H_2_S gas generation when they are exposed to air. Sahu *et al.* reported that the stability of sulfide solid electrolytes to moisture in air depends on the hard and soft acids and bases (HSAB) theory.^10^ Based on the HSAB theory, sulfide solid electrolytes with soft acids such as Sb, As, and Sn show higher air stability than those with hard acids such as P because the former prevent the replacement of sulfur (soft base) in solid electrolytes with oxygen (hard base) in air. Li_4_SnS_4_-based solid electrolytes have been especially investigated due to their relatively high ionic conductivity and high air stability.^10–18^

Kaib *et al.* reported that orthorhombic Li_4_SnS_4_ prepared by solid-phase synthesis showed ionic conductivity of 7 × 10^−5^ S cm^−1^ at 20 °C.^11^ Ionic conductivity of orthorhombic Li_4_SnS_4_ increases by the partial substitution of group 15 elements for Sn. Li_3.833_Sn_0.833_As_0.166_S_4_ (ref. 10) and Li_3.85_Sn_0.85_Sb_0.15_S_4_ (ref. 16) prepared by solid-phase syntheses exhibit ionic conductivities of 1.39 × 10^−3^ S cm^−1^ at 25 °C and 8.5 × 10^−4^ S cm^−1^ at 30 °C, respectively. In contrast, the substitution of group 13 elements such as Ga and Al results in low ionic conductivity of 10^−6^ S cm^−1^.^19,20^ In solid-phase synthesis, only a few toxic elements such as As and Sb enhance the ionic conductivity of Li_4_SnS_4_.

Park *et al.* reported that 0.4LiI·0.6Li_4_SnS_4_ glass can be synthesized by dissolving LiI and orthorhombic Li_4_SnS_4_ in ethanol and *via* subsequent heat treatment.^12^ Furthermore, 0.4LiI·0.6Li_4_SnS_4_ glass has an ionic conductivity of 4.1 × 10^−4^ S cm^−1^ at 30 °C and high deformability. As Li_4_SnS_4_ shows high stability to moisture, Choi *et al.* coated Li_4_SnS_4_ on LiCoO_2_ prepared from aqueous solution^13^ and Matsuda *et al.* synthesized Li_4_SnS_4_ by ion-exchange of Na_4_SnS_4_ aqueous solution.^18^

Hexagonal Li_4_SnS_4_ reported as the metastable phase is synthesized by mechanochemical treatment of Li_2_S and SnS_2_.^14^ Sintered bodies of hexagonal Li_4_SnS_4_ showed a higher ionic conductivity of 1.1 × 10^−4^ S cm^−1^ at 25 °C than orthorhombic Li_4_SnS_4_ prepared by solid-phase synthesis. To further increase the ionic conductivity, we focused on the addition of other components to hexagonal Li_4_SnS_4_ by mechanochemical treatment.

In the present study, *x*LiI·(1 − *x*)Li_4_SnS_4_ solid electrolytes were prepared by mechanochemical treatment. Addition of LiI increases ionic conductivity and formability, as has been reported in Li_2_S–P_2_S_5_ systems.^21–25^ While *x*LiI·(1 − *x*)Li_4_SnS_4_ glass was obtained by liquid-phase synthesis,^12^ we found that the crystal phase was obtained by mechanochemical treatment. To further improve the ionic conductivity without reducing the stability of the Li_4_SnS_4_-based solid electrolytes under air, LiI–Li_3_PS_4–_Li_4_SnS_4_ solid electrolytes were prepared. Li_3_PS_4_ was selected since it exhibits the highest air stability in Li_2_S–P_2_S_5_ systems because it consists of only PS_4_ tetrahedra.^26^ In addition, Li_3_PS_4_ glass was selected because it shows a relatively high ionic conductivity (∼4 × 10^−4^ S cm^−1^ at 25 °C) compared to other Li_2_S–P_2_S_5_ systems.^27^ In addition, the air stabilities of the prepared solid electrolytes were compared. The purpose of the present study was to develop novel hexagonal Li_4_SnS_4_-based solid electrolytes with both high air stability and ionic conductivity through mechanochemical treatment.

## Experimental

### Preparation of solid electrolytes

We prepared *x*LiI·(1 − *x*)Li_4_SnS_4_ (*x* = 0, 0.40–0.50), *y*Li_3_PS_4_·(1 − *y*)Li_4_SnS_4_ (*y* = 0–0.5) and *z*LiI·(1 − *z*)(0.4Li_3_PS_4_·0.6Li_4_SnS_4_) (*z* = 0.37–0.50) solid electrolytes by mechanochemical treatment. The mixtures (0.5 g) of Li_2_S (99.9%; Mitsuwa Chemical Co., Ltd), SnS_2_ (99.5%; Mitsuwa Chemical Co., Ltd), LiI (ultra dry, 99.999%; Alfa Aesar), and P_2_S_5_ (Merck) were placed in a ZrO_2_ pot (45 mL) with ZrO_2_ balls (4 mmΦ, 90 g) in a dry Ar atmosphere. They were mechanically milled at 510 rpm for 10–60 h using a planetary ball mill apparatus (Pulverisette 7; Fritsch). Furthermore, the samples with *x* = 0, 0.43, *z* = 0.37, and 0.43 were heated at 390 °C, 200/270 °C, 200/240 °C, and 230 °C, respectively, for 2 h in an Ar atmosphere to obtain heat-treated samples. Li_5.5_PS_4.5_Cl_1.5_ solid electrolytes were prepared by mechanochemical treatment of Li_2_S, P_2_S_5_, and LiI (99.9%; Kojundo Chemical Laboratory Co., Ltd). The starting materials are put in a ZrO_2_ pot (250 mL) with 12 ZrO_2_ balls (20 mmΦ) and milled at 330 rpm for 30 h using a planetary ball mill apparatus (Pulverisette 5; Fritsch). Li_3_PS_4_ glass was prepared by mechanochemical treatment of Li_2_S and P_2_S_5_ with heptane and dibutyl ether. The details are in the previous reports.^28^

### Characterization of solid electrolytes

XRD measurements were conducted for the prepared solid electrolytes using an X-ray diffractometer (D8 ADVANCE; Bruker AXS) with Cu Kα radiation. Furthermore, synchrotron XRD measurements were performed for the samples with *x* = 0, 0.40 and 0.43 in *x*LiI·(1 − *x*)Li_4_SnS_4_ at the BL19B2 beamline of SPring-8 at room temperature (∼25 °C) with a wavelength of 0.5002 Å to obtain their crystallographic data. The samples were sealed in quartz capillaries (∼0.3 mm in diameter) in a dry Ar atmosphere. Rietveld refinement was performed using the RIETAN-FP program.^29^ In addition, pair distribution function (PDF) analysis was conducted for the samples with *x* = 0 and 0.43 using an X-ray diffractometer (SmartLab; Rigaku Corp.) with Mo Kα radiation.

Raman spectroscopy was performed for the samples with *x* = 0 and 0.43 using a Raman microscope (inVia Raman Microscope; RENISHAW) with a green laser (532 nm) and a 50× objective lens (NA = 0.50, Leica Microsystems).

We plotted DSC curves of the samples with prepared solid electrolytes using a thermal analyzer (DSC-60 Plus; Simadzu Corp.). The samples were sealed with an Al pan in a dry Ar atmosphere and heated up to 500 °C from room temperature at a heating rate of 10 °C min^−1^.

To evaluate the ionic conductivity of the prepared solid electrolytes, AC impedance measurements were performed with an applied voltage of 50 mV and a frequency range of 10 Hz–10 MHz using an impedance analyzer (1260A; Solartron Analytical). The samples were pelletized to a diameter of 10 mm at 360 MPa for 5 min. To measure the sample with *z* = 0.37 in *z*LiI·(1 − *z*)(0.4Li_3_PS_4_·0.6Li_4_SnS_4_) heated at 200 and 240 °C, pelletized samples were heated to obtain sintered bodies. The samples were sandwiched with stainless steel (SUS) rods as current collectors.

For cyclic voltammetry (CV) measurements, an asymmetric cell (Li/solid electrolyte/SUS) was prepared. The solid electrolyte layer was prepared by uniaxially pressing at 360 MPa for 5 min. The Li foil (8 mm in diameter) was attached to the solid electrolyte layer and pressed at 72 MPa for 2 min as a reference and a counter electrode. The other side of the solid electrolyte layer was directly attached to the SUS rod (working electrode). CV measurements were conducted for the cells with a potential sweep varying from −0.5 to 5.0 V and a scanning rate of 1 mV s^−1^ at 25 °C using an electrochemical measurement device (Celltest System 1470 E; Solartron Analytical).

### Construction of all-solid-state cells

All-solid-state cells were constructed with composite positive electrodes consisting of the solid electrolytes with *x* = 0, 0.43 or *z* = 0.37 (heated at 200 °C) and a LiNi_1/3_Mn_1/3_Co_1/3_O_2_ (NMC) positive electrode to compare battery performance. The composite positive electrodes were prepared by mixing solid electrolytes and LiNbO_3_-coated NMC at a weight ratio of 30/70. LiNbO_3_ was used as the buffer layer because it can reduce the interfacial resistance between sulfide solid electrolytes and NMC.^30^ LiNbO_3_ coating was prepared by spray coating of an ethanol solution of lithium niobium ethoxide and subsequent heat treatment at 400 °C for 30 min under O_2_ flow.^28,30^ The Li_3_PS_4_ glass solid electrolyte (80 mg) was pressed at 36 MPa to prepare a solid electrolyte layer. The composite positive electrode (10 mg) was placed on the solid electrolyte layer and uniaxially pressed at 360 MPa for 5 min. An indium foil (99.999%; 0.1 mm^*t*^; Furuuchi Chemical Corp.) (*ϕ* = 9 mm) and a lithium piece (99.9%, 0.2 mm^*t*^; Honjo Metal Co., Ltd) (1 mg) were placed on the opposite sides and pressed at 180 MPa for 2 min. The cells (diameter in 10 mm) were sandwiched with the SUS rods and operated at 30 °C with a cut-off voltage of 2.4–3.6 V (*vs.* Li–In) at 0.1, 0.2, 0.5 and 1C rates (1C = 0.9–1.0 mA) using a charge–discharge measuring device (ABE 1024-5V; Electro Field Co., ltd).

### Air stability test of solid electrolytes

To evaluate the air stability of the solid electrolytes, H_2_S gas generation was monitored in a sealed chamber (2000 mL) with a relative humidity of 50% at 25 °C. The solid electrolyte powder (50 mg), a small fan, and an H_2_S gas sensor (Gasman; Crowcon Detection Instruments Ltd) were placed in the chamber. The amount of H_2_S gas (ppm) was monitored by the H_2_S gas sensor. The generated H_2_S gas volume (*V*; cm^3^ g^−1^) was calculated using the following equation: *V* = (*C* × *L* × 10^−6^)/*m*,^14^ where *C* is the concentration of H_2_S gas (ppm), *L* is the volume of the chamber (cm^3^), and *m* is the weight of the solid electrolyte powder (g). Structures of air-exposed samples were examined by XRD measurements. The samples were exposed in air at 50% RH for 3 min, then sealed in an XRD holder in air, and measured for 60 min.

## Results and discussion

### Preparation and characterization of solid electrolytes in the LiI–Li_4_SnS_4_ system

**Fig. 1:**
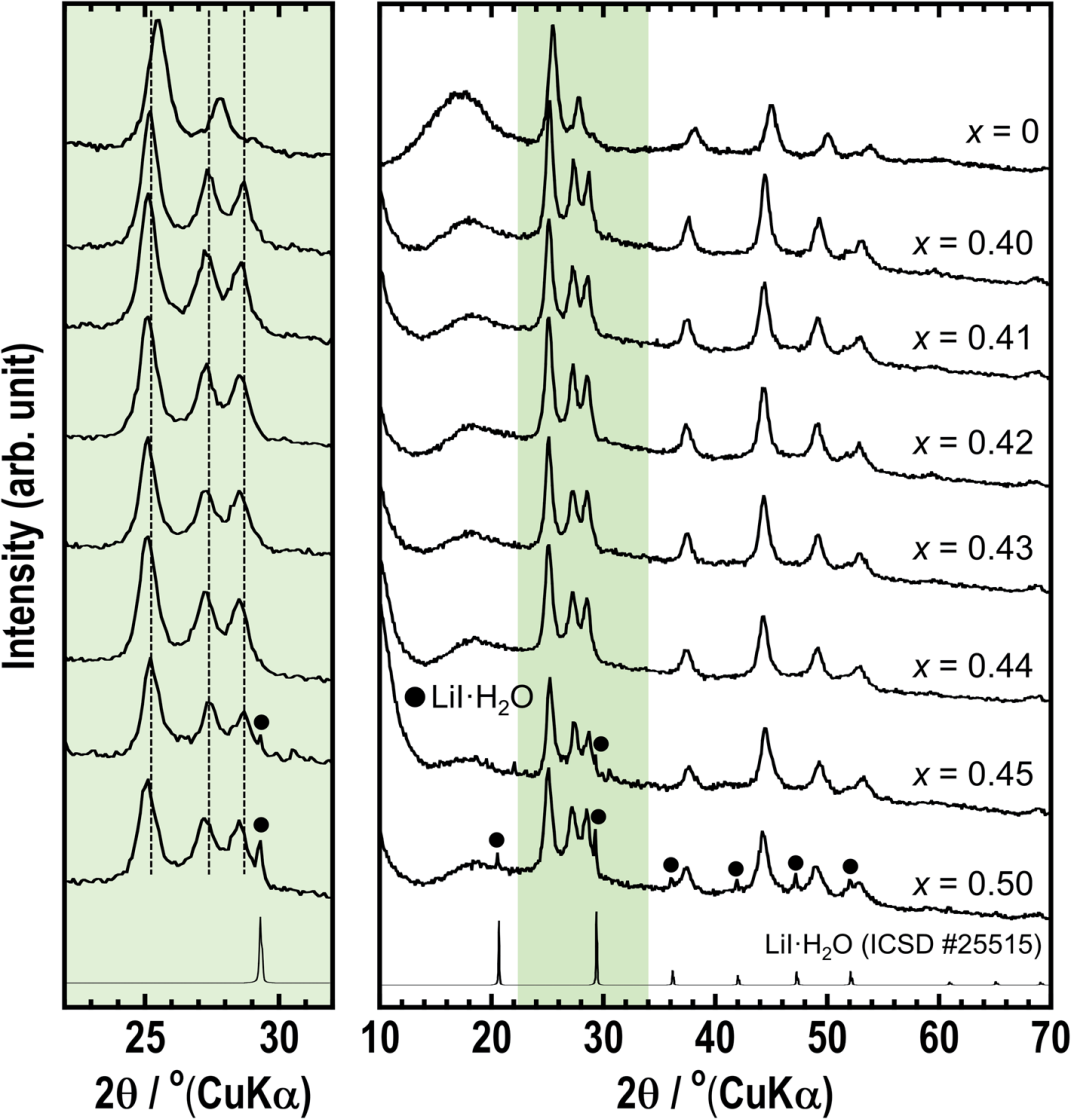
XRD patterns of *x*LiI·(1 − *x*)Li_4_SnS_4_ (*x* = 0, 0.40–0.50). The left part is the enlarged XRD pattern between 22–32°.

The structures of the prepared *x*LiI·(1 − *x*)Li_4_SnS_4_ (*x* = 0 and 0.40 ≤ *x* ≤ 0.50) solid electrolytes were examined by XRD measurements (Fig. 1). The diffraction peaks of hexagonal Li_4_SnS_4_ observed at *x* = 0 shifted to a lower angle after the addition of LiI because I^−^ with a large ionic radius was introduced into the structure. In addition, a new peak appeared at approximately 28–29°. The XRD simulation (not shown here) showed that the intensity of the peak at 28–29° increased with increasing Li/Sn occupation ratio. Therefore, the new peak may be attributed to the increase in the Li/Sn ratio due to the addition of LiI. The XRD patterns of the *x* = 0.45 and *x* = 0.50 samples showed small diffraction peaks of LiI·H_2_O (ICSD #25515), indicating that LiI could not dissolve in Li_4_SnS_4_ completely in the case of more than *x* = 0.45.

The *x*LiI·(1 − *x*)Li_4_SnS_4_ solid electrolytes can be prepared from not only Li_2_S, SnS_2_, and LiI but also from hexagonal or orthorhombic Li_4_SnS_4_ and LiI as starting materials. Fig. S1 in the ESI† shows XRD patterns of 0.4LiI·0.6Li_4_SnS_4_ prepared from LiI and hexagonal Li_4_SnS_4_ or orthorhombic Li_4_SnS_4_, which were prepared by heat treatment of hexagonal Li_4_SnS_4_. These 0.4LiI·0.6Li_4_SnS_4_ samples showed similar XRD patterns as the 0.4LiI·0.6Li_4_SnS_4_ sample prepared from Li_2_S, SnS_2_, and LiI. Moreover, we also confirmed that orthorhombic Li_4_SnS_4_ prepared by the solid-phase synthesis of Li_2_S and SnS_2_ could be used as a starting material of *x*LiI·(1 − *x*)Li_4_SnS_4_ solid electrolytes (not shown here).

**Fig. 2:**
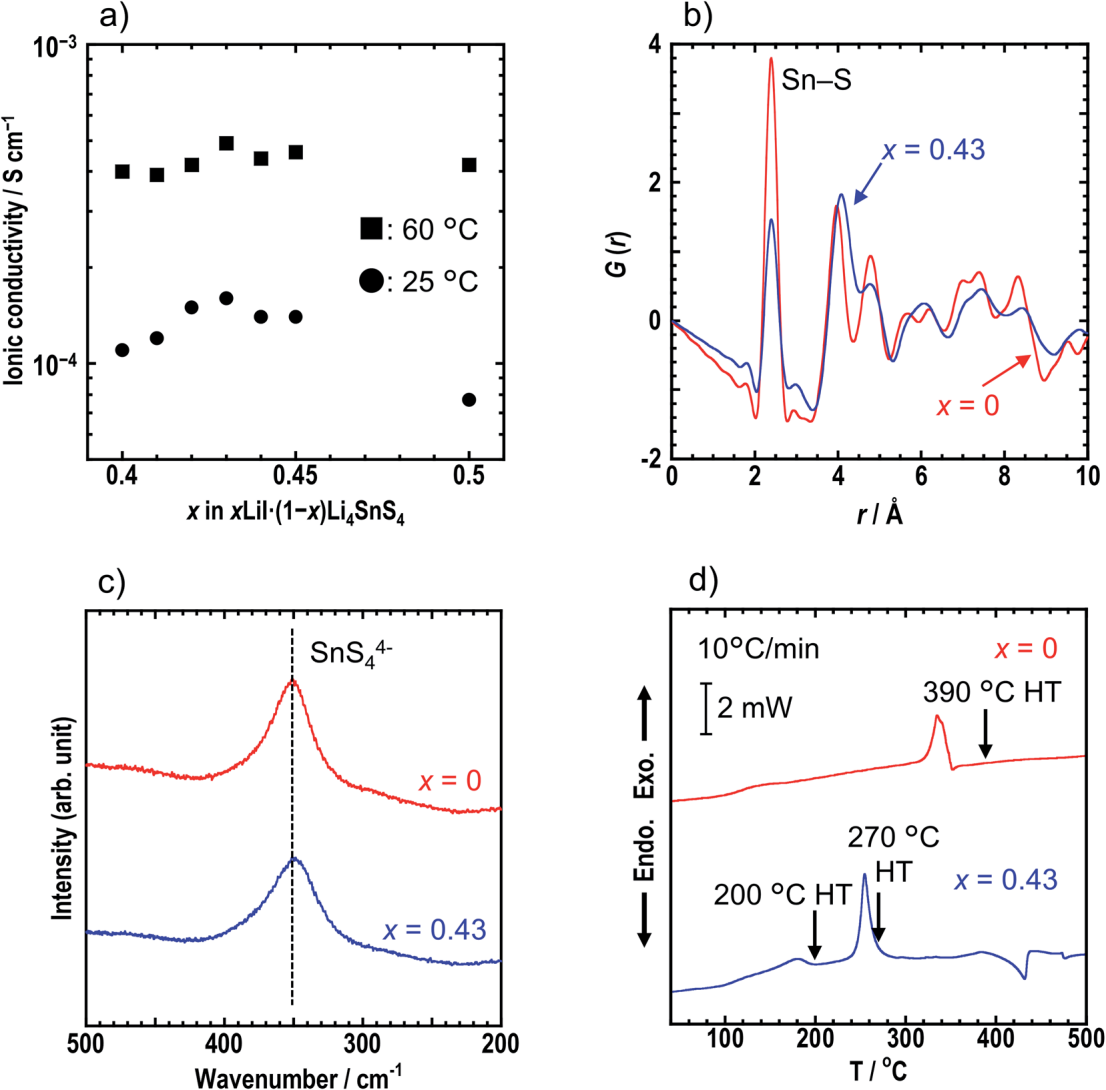
(a) The ionic conductivities of *x* = 0.40–0.50 in *x*LiI·(1 − *x*)Li_4_SnS_4_ at 25 °C and 60 °C. (b) *G*(*r*) and (c) Raman spectra of *x* = 0 and 0.43 in *x*LiI·(1 − *x*)Li_4_SnS_4_. The Sn–S correlation in the SnS_4_ tetrahedra corresponds to *r* = 2.4 Å. (d) DSC curves of *x* = 0 and 0.43. The heating rate was 10 °C min^−1^. The arrows indicate heat treatment (HT) temperatures for heat-treated samples.

Ionic conductivities of cold-pressed *x*LiI·(1 − *x*)Li_4_SnS_4_ were measured by the AC impedance technique (Table S1†). Fig. S2† shows the Nyquist plots of the sample with *x* = 0.43 at 25 °C. The conductivity was determined from the total resistance (*R*_total_ = *R*_b_ + *R*_g.b_) because the resistances of bulk (*R*_b_) and grain boundaries (*R*_g.b._) could not be distinguished. Addition of LiI to Li_4_SnS_4_ enhanced their ionic conductivity (∼1 × 10^−4^ S cm^−1^ at 25 °C) compared with Li_4_SnS_4_ (4.5 × 10^−5^ S cm^−1^ at 25 °C). The ionic conductivities of *x* = 0.40–0.50 in *x*LiI·(1 − *x*)Li_4_SnS_4_ at 25 °C and 60 °C are plotted in Fig. 2a. The sample where *x* = 0.43 exhibited the highest ionic conductivity of 1.6 × 10^−4^ S cm^−1^ at 25 °C due to the greater amount of dissolved LiI, whereas at *x* = 0.50, the ionic conductivity was reduced due to the remaining LiI.

We also prepared Li_2_S–SnS_2_ solid electrolytes with Li_2_S/SnS_2_ amounts of 70/30 and 80/20 (mol%). Note that the ratio of 67/33 indicates Li_4_SnS_4_. The XRD patterns of the 70/30 and 80/20 solid electrolytes indicated that Li_2_S remained (Fig. S3a†), and their ionic conductivities were lower than those of the 67/33 sample (Table S2†). Therefore, increasing the amount of Li_2_S in the Li_2_S–SnS_2_ system is not an effective way to increase the Li carrier and ionic conductivity. Furthermore, addition of LiI to the 70/30 and 80/20 solid electrolytes increased their ionic conductivities—7.3 × 10^−5^ and 5.8 × 10^−5^ S cm^−1^, respectively at 25 °C—although they were lower than that of the 67/33 solid electrolyte with LiI (Fig. S3b and Table S2†).

We focused on *x* = 0.43 in *x*LiI·(1 − *x*)Li_4_SnS_4_ exhibiting the highest ionic conductivity hereafter. Synchrotron XRD, PDF analysis, and Raman spectroscopy were conducted for the *x* = 0 and *x* = 0.43 samples to compare their structures in detail. Fig. S4† shows the synchrotron XRD patterns and structures of the *x* = 0, 0.40, and 0.43 samples characterized by Rietveld refinement. Crystallographic data of *x* = 0, 0.40, and 0.43 are shown in Tables S3, S4, and S5,† respectively. All samples were determined as *P*6_3_/*mmc* space group irrespective of the presence of LiI. The samples with LiI were refined by partial replacement of S by I. The lattice volume increased with the addition of LiI, suggesting that I^−^, which has a large radius, was introduced into the structure and that LiI-added Li_4_SnS_4_ formed solid solutions. The occupancies of Li were not determined in the present study. Rietveld analysis was conducted for both *x* = 0.40 and 0.43 with an Li occupation rate of 0.375 based on a previous report on Li_4_SnS_4_.^14^ To investigate the local structure, PDF analyses were conducted for *x* = 0 and *x* = 0.43 samples using a laboratory X-ray diffractometer with Mo Kα radiation. The *G*(*r*) of *x* = 0 and 0.43 samples are shown in Fig. 2b. Both samples showed the first peak at ∼2.1 Å, corresponding to the Sn–S correlation in the SnS_4_ tetrahedra.^11^ In addition, both samples showed a Raman band at 350 cm^−1^ which was attributed to the SnS_4_^4−^ unit (Fig. 2c).^31^ Note that the Raman band of the *x* = 0.43 sample is slightly broader than that of the *x* = 0 sample. The results of the first peak in PDF analysis and the Raman spectra suggest that the SnS_4_ tetrahedra mostly remain unchanged by the addition of LiI. However, the second peak of *x* = 0.43 in the PDF analysis is observed at a longer distance than that of *x* = 0, suggesting that iodine exists outside of the SnS_4_ tetrahedra but near S in the SnS_4_ tetrahedra. From the Faber–Ziman coefficient in *x* = 0.43, the *w*_*ij*_ values of the S–S and S–I correlations are 0.145 and 0.18, respectively, indicating that it is hard to distinguish between S–S and S–I bonds. Regarding previous studies on PDF analysis of LiI-added Li_3_PS_4_, Takahashi *et al.* reported that iodine was inserted between PS_4_ anions in the 70Li_3_PS_4_·30LiI (mol%) solid electrolytes because it showed no peak from the P–I bond in the exchanged S and I model.^32^ Similarly, in the case of Li_4_SnS_4_, iodine was assumed to exist between the SnS_4_ anion, while Li_4_SnS_4_ with LiI was refined by partial replacement of S by I, as observed in the XRD results. Further refinement by maximum entropy methods will reveal new site of iodine in the structure. The results of the XRD, PDF, and Raman measurements suggest that iodine was introduced between SnS_4_ tetrahedra and caused expansion of diffusion paths of Li^+^, resulting in higher ionic conductivity.

Thermal behaviors of *x* = 0 and *x* = 0.43 were examined by DSC measurements (Fig. 2d). The DSC curve of *x* = 0.43 shows two exothermic peaks at 180 °C and 260 °C, whereas the *x* = 0 sample has a peak at 340 °C. Hexagonal and orthorhombic Li_4_SnS_4_ were obtained by heat treatment of as-milled Li_4_SnS_4_ at 260 °C and 390 °C, respectively.^14^ Fig. S5† shows XRD patterns of *x* = 0 and *x* = 0.43 samples before and after heat treatment. In the present study, orthorhombic Li_4_SnS_4_ was obtained by heat treatment at 390 °C, as reported. The crystallinity of the *x* = 0.43 sample increased by heating at 200 °C. Furthermore, the XRD pattern of *x* = 0.43 heated at 270 °C exhibited specific diffraction peaks attributable to orthorhombic Li_4_SnS_4_ and LiI·H_2_O, especially at 10°–24°. In the case of Li_4_SnS_4_ with LiI, transition to orthorhombic Li_4_SnS_4_ occurred at lower temperature compared with Li_4_SnS_4_, suggesting that Li_4_SnS_4_ with LiI is thermodynamically metastable. Therefore, preparing them through high temperature synthesis such as conventional solid-phase reaction is challenging.

### Preparation and characterization of solid electrolytes in the LiI–Li_3_PS_4_–Li_4_SnS_4_ system

To increase the ionic conductivity of *x*LiI·(1 − *x*)Li_4_SnS_4_ solid electrolytes, we considered addition of Li_3_PS_4_, which shows higher ionic conductivity than Li_4_SnS_4_. In advance, we determined the best ratio within *y* = 0–0.5 (*y*Li_3_PS_4_·(1 − *y*)Li_4_SnS_4_). We reported a systematic study on ionic conductivities and structures of *y*Li_3_PS_4_·(1 − *y*)Li_4_SnS_4_.^33^ Fig. S6† shows the XRD patterns and ionic conductivities at 25 and 60 °C of *y* = 0–0.5 in *y*Li_4_SnS_4_·(1 − *y*)Li_3_PS_4_. As the ratio of Li_3_PS_4_ was increased, the intensity of the diffraction peaks of hexagonal Li_4_SnS_4_ decreased, and ionic conductivity increased. Compared with *y* = 0.4 and 0.5, both samples showed similar ionic conductivity. We determined *y* = 0.4 is the best ratio, which is expected to show higher air stability, due to the excess Li_4_SnS_4_ than the *y* = 0.5 sample.

**Fig. 3:**
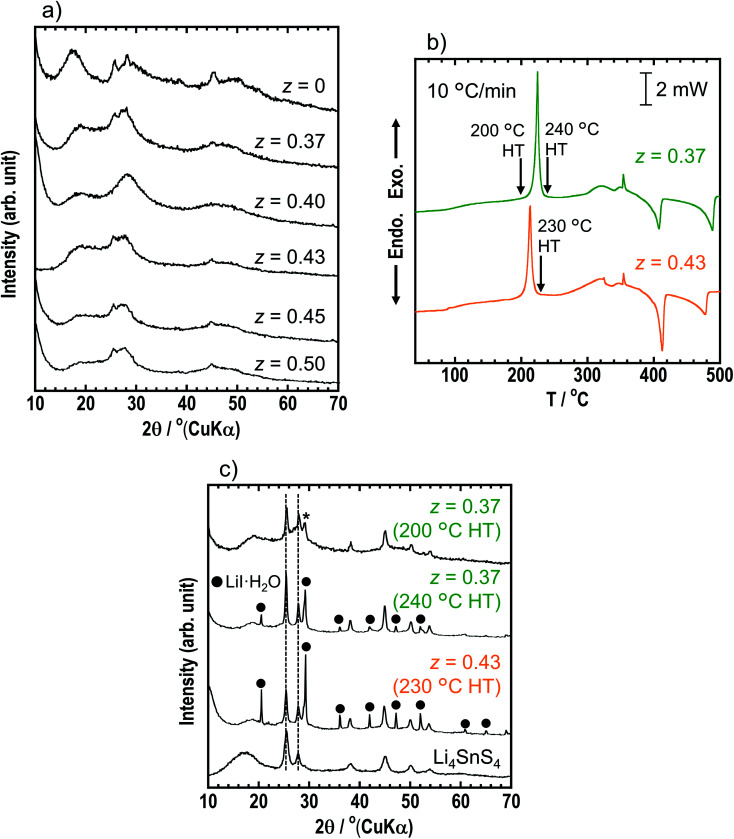
(a) XRD patterns of *z* = 0, 0.37, 0.40, 0.43, 0.45 and 0.50 in as-milled *z*LiI·(1 − *z*)(0.4Li_3_PS_4_·0.6Li_4_SnS_4_). (b) DSC curves of *z* = 0.37 and 0.43. The heating rate was 10 °C min^−1^. The arrows indicated heat treatment (HT) temperatures for heat-treated samples. (c) XRD patterns of *z* = 0.37 and 0.43 heated at 200/240 °C and 230 °C, respectively and Li_4_SnS_4_.

The *z*LiI·(1 − *z*)(0.4Li_3_PS_4_·0.6Li_4_SnS_4_) solid electrolytes were prepared by mechanochemical treatment using Li_2_S, SnS_2_, P_2_S_5_, and LiI as starting materials. Fig. 3a shows the XRD patterns of as-milled solid electrolytes with *z* = 0, 0.37, 0.40, 0.43, 0.45, and 0.50. The XRD patterns of the samples with LiI exhibited halo patterns and small diffraction peaks derived from hexagonal Li_4_SnS_4_ at 25.4° and 45.1°, suggesting that LiI–Li_3_PS_4_–Li_4_SnS_4_ systems can easily form the amorphous state due to its various components. The *z* = 0.37 sample consisted of 0.37LiI·0.25Li_3_PS_4_·0.38Li_4_SnS_4_, and it was prepared with the highest amount of Li_4_SnS_4_ in Li_4_SnS_4_-based *z*LiI·(1 − *z*)(0.4Li_3_PS_4_·0.6Li_4_SnS_4_). In the *z* ratio of more than 0.37, the amount of LiI was higher than that in Li_4_SnS_4_. In the case of *x*LiI·(1 − *x*)Li_4_SnS_4_, diffraction peaks attributed to residual LiI were observed in the XRD patterns of *x* = 0.45 and 0.50 samples (Fig. 1). In contrast, the diffraction peaks of LiI were not observed in *z* = 0.40–0.50, suggesting that excess LiI reacted in *z*LiI·(1 − *z*)(0.4Li_3_PS_4_·0.6Li_4_SnS_4_). DTA measurements were performed for *z* = 0.37 and 0.43 (Fig. 3b). The DTA curves of *z* = 0.37 and 0.43 show large exothermic peaks at 220 and 210 °C, respectively, and two endothermic peaks between 400 and 500 °C. The *z* = 0.37 sample was heated at 200 and 240 °C, and *z* = 0.43 was heated at 230 °C. The XRD patterns of *z* = 0.37 and 0.43 after heat treatment are shown in Fig. 3c. The *z* = 0.37 sample heated at 200 °C has a hexagonal Li_4_SnS_4_ phase with a diffraction peak at 28–29° indicated by an asterisk, which exhibits a high Li/Sn ratio, as mentioned before. Furthermore, the peaks originated from hexagonal Li_4_SnS_4_ did not shift. In contrast, *x*LiI·(1 − *x*)Li_4_SnS_4_ showed peak-shifting to the lower angle, as shown in Fig. 1. In addition, in our previous report, Li_4_SnS_4_ with added Li_3_PS_4_ showed slight peak-shifting to a higher angle.^33^ These behaviors suggested that Li_4_SnS_4_ with LiI and Li_3_PS_4_ tended not to exhibit peak-shifting. Therefore, *z* = 0.37 heated at 200 °C enhanced the Li/Sn ratio compared with Li_4_SnS_4_ without decomposition reactions. In contrast, after heat treatment at 240 °C, diffraction peaks of LiI·H_2_O were observed, suggesting that the exothermic peak at ∼220 °C in the DSC curve corresponded to the decomposition reaction. In the *z* = 0.43 sample, after heat treatment at 230 °C, the intensity of the peaks attributable to LiI·H_2_O increased even though it was heated at a lower temperature, indicating that the decomposition reaction could easily proceed with the large amount of LiI.

**Fig. 4:**
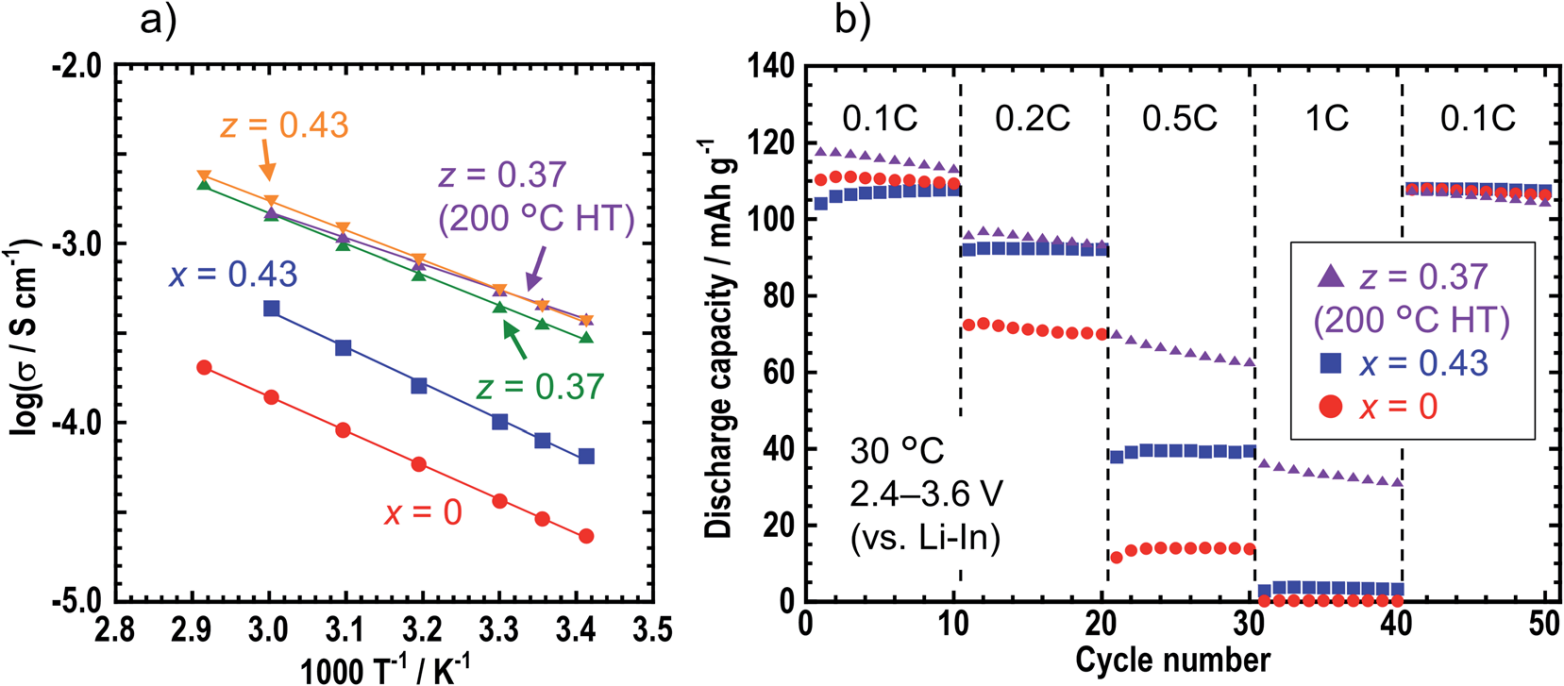
(a) Temperature dependence of ionic conductivities of *x* = 0 and *x* = 0.43 samples in *x*LiI·(1 − *x*)Li_4_SnS_4_ and *z* = 0.37 (before and after the heat treatment at 200 °C) and *z* = 0.43 in *z*LiI·(1 − *z*)(0.4Li_3_PS_4_·0.6Li_4_SnS_4_). (b) Discharge capacities of the all-solid-state cells where *x* = 0, *x* = 0.43 and *z* = 0.37 (heating at 200 °C) at rates of 0.1, 0.2, 0.5, and 1C.

Table 1 shows the ionic conductivities of the *z* = 0, 0.37, 0.40, 0.43, and 0.50 samples in *z*LiI·(1 − *z*)(0.4Li_3_PS_4_·0.6Li_4_SnS_4_) at room temperature (25 °C) before and after heat treatment. The ionic conductivities of the LiI-added samples of *z* = 0.37, 0.40, 0.43, and 0.50 before heat treatment increased up to *ca.* 5 × 10^−4^ S cm^−1^ compared with those of *z* = 0. The ionic conductivity of *z* = 0.37 heated at 200 °C was not different from that of the sample before heat treatment, while that of *z* = 0.37 heated at 240 °C decreased to 1.5 × 10^−4^ S cm^−1^ because of decomposition. Fig. 4 shows the temperature dependence of the ionic conductivities of *x* = 0 and 0.43 in *x*LiI·(1 − *x*)Li_4_SnS_4_ and *z* = 0.37 (before and after the heat treatment at 200 °C) and 0.43 in *z*LiI·(1 − *z*)(0.4Li_3_PS_4_·0.6Li_4_SnS_4_). These ionic conductivities obeyed Arrhenius law of *σ* = *σ*_0_ exp(−*E*_a_/*RT*), where *σ*_0_ is a pre-exponential factor, *E*_a_ is an activation energy, *R* is the gas constant, and *T* is temperature. The activation energies were calculated by slopes of the Arrhenius plots. The ionic conductivities at 30 °C and the activation energies of these samples are listed in Table S6† with the data of 0.4LiI·0.6Li_4_SnS_4_ glass.^12^ The activation energies of the LiI–Li_3_PS_4_–Li_4_SnS_4_ systems before and after heat treatment were 32–33 and 28 kJ mol^−1^, respectively. The *z* = 0.37 sample heated at 200 °C showed the lowest activation energy, whereas the LiI–Li_4_SnS_4_ systems showed higher activation energies of nearly 40 kJ mol^−1^. Furthermore, *z* = 0.37 and 0.43 samples exhibited higher ionic conductivities at 30 °C (5.5 × 10^−4^ S cm^−1^) than 0.4LiI·0.6Li_4_SnS_4_ glass prepared by the liquid-phase synthesis.^12^ Therefore, *z*LiI·(1 − *z*)(0.4Li_3_PS_4_·0.6Li_4_SnS_4_) solid electrolytes prepared by mechanochemical treatment exhibited higher ionic conductivity and lower activation energy than those reported for LiI-added Li_4_SnS_4_-based solid electrolytes.

**Table 1:** Ionic conductivities of *z*LiI·(1 − *z*)(0.4Li_3_PS_4_·0.6Li_4_SnS_4_) at 25 °C. “200 °C HT” and “240 °C HT” are indicated after heat treatment at 200 °C and 240 °C, respectively

	LiI : Li_3_PS_4_ : Li_4_SnS_4_ (mol%)	*σ*_25 °C_/S cm^−1^
*z* = 0 (before HT)	0 : 40 : 60	2.5 × 10^−4^
*z* = 0.37 (before HT)	37 : 25 : 38	4.5 × 10^−4^
*z* = 0.37 (200 °C HT)	37 : 25 : 38	4.6 × 10^−4^
*z* = 0.37 (240 °C HT)	37 : 25 : 38	1.5 × 10^−4^
*z* = 0.40 (before HT)	40 : 24 : 36	5.0 × 10^−4^
*z* = 0.43 (before HT)	43 : 23 : 34	5.1 × 10^−4^
*z* = 0.50 (before HT)	50 : 20 : 30	4.9 × 10^−4^

To investigate the electrochemical stability windows of the Li_4_SnS_4_-based solid electrolytes, cyclic voltammetry was conducted for the Li/solid electrolytes/SUS asymmetric cells using Li_4_SnS_4_ (*x* = 0), 0.43LiI·0.57Li_4_SnS_4_ (*x* = 0.43 in *x*LiI·(1 − *x*)Li_4_SnS_4_), and 0.37LiI·0.25Li_3_PS_4_·0.38Li_4_SnS_4_ with heating at 200 °C (*z* = 0.37 in *z*LiI·(1 − *z*)(0.4Li_3_PS_4_·0.6Li_4_SnS_4_)) as solid electrolyte layers (Fig. S7†). The cyclic voltammograms of all cells exhibited several peaks between −0.5 and 3 V (*vs.* Li^+^/Li), in addition to a pair of reduction and oxidation peaks close to 0 V, which corresponded to lithium deposition and reduction. This indicates that Li_4_SnS_4_-based solid electrolytes undergo decomposition between −0.5 and 3 V (*vs.* Li^+^/Li). However, in Fig. S7,† no peaks were observed between 3 and 5 V, thereby suggesting that the Li_4_SnS_4_-based solid electrolytes are stable at high voltages and can be used in a composite positive electrode layer. Moreover, we note that in our previous report, Li_4_SnS_4_ was found to exhibit a high thermal stability to oxide positive electrodes.^34^

Charge–discharge tests were carried out for the all-solid-state cells containing NMC composite positive electrodes with *x* = 0, *x* = 0.43, and *z* = 0.37 (heated at 200 °C) solid electrolytes. Fig. 4b shows the discharge capacities of the cells at 0.1, 0.2, 0.5, and 1C. In the case where the *z* = 0.37 sample was used in the composite positive electrode, the rate performance improved compared with those obtained for the *x* = 0 and 0.43 samples because of the higher ionic conductivity of the *z* = 0.37 sample.

### Air stability tests of the prepared solid electrolytes

**Fig. 5:**
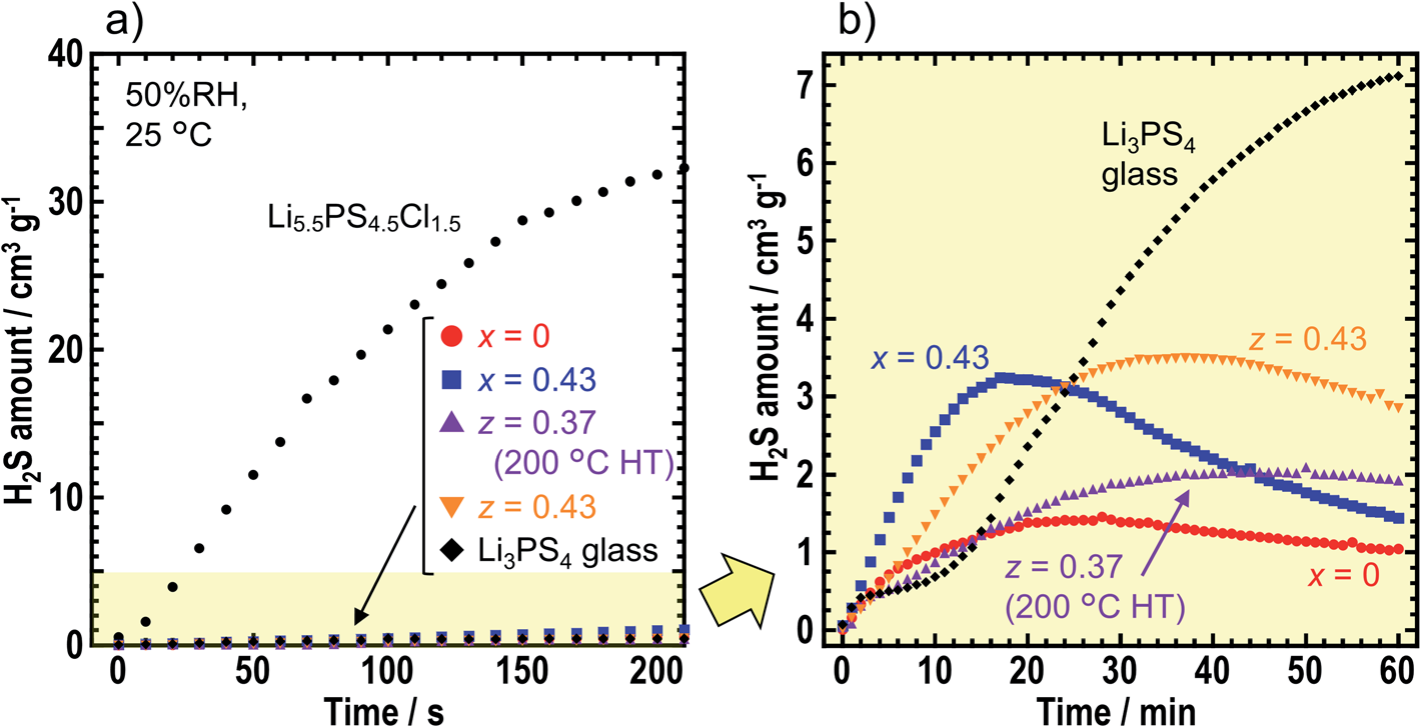
(a) H_2_S generation when the solid electrolyte powder was sealed in a desiccator filled with air (50% RH) at 25 °C for 210 s. The following samples were examined: Li_5.5_PS_4.5_Cl_1.5_, Li_3_PS_4_ glass, *x* = 0 and *x* = 0.43 in *x*LiI·(1 − *x*)Li_4_SnS_4_ and *z* = 0.37 (heating at 200 °C) and *z* = 0.43 in *z*LiI·(1 − *z*)(0.4Li_3_PS_4_·0.6Li_4_SnS_4_). The amount of solid electrolyte employed was 2 mg for Li_5.5_PS_4.5_Cl_1.5_ and 50 mg for all others. (b) H_2_S generation from the various solid electrolytes with the exception of Li_5.5_PS_4.5_Cl_1.5_. The measurement time was 60 min.

To compare air stabilities of the prepared solid electrolytes, H_2_S gas generation was monitored when the solid electrolyte powder was placed in the desiccator filled with air (50% RH). Fig. 5 shows the amount of H_2_S gas generated by *x* = 0 and *x* = 0.43 in *x*LiI·(1 − *x*)Li_4_SnS_4_, and *z* = 0.37 (after the heat treatment at 200 °C) and *z* = 0.43 in *z*LiI·(1 − *z*)(0.4Li_3_PS_4_·0.6Li_4_SnS_4_). We compared Li_3_PS_4_ glass and an argyrodite-type solid electrolyte of Li_5.5_PS_4.5_Cl_1.5_. In Fig. 5a, 2 mg of Li_5.5_PS_4.5_Cl_1.5_ emitted the largest amount of H_2_S in 210 s although the amount of Li_5.5_PS_4.5_Cl_1.5_ was 1/25 of that other solid electrolytes. Therefore, argyrodite-type solid electrolytes show extremely low air stability compared with Li_4_SnS_4_-based solid electrolytes and Li_3_PS_4_ glass.

Fig. 5b shows the amount of H_2_S gas generated from the Li_4_SnS_4_-based solid electrolytes and Li_3_PS_4_ glass in 1 h. Note that the range of H_2_S amount changed from 0–40 to 0–5 cm^3^ g^−1^. With an exposure time of 60 min, Li_3_PS_4_ glass emitted the highest amount of H_2_S gas over the various solid electrolytes, thereby indicating that hexagonal Li_4_SnS_4_-based solid electrolytes exhibited higher air stabilities than Li_3_PS_4_ glass. The Li_4_SnS_4_ with LiI tended to emit higher amount of H_2_S gas than Li_4_SnS_4_. For the first 10–15 min, the speed of H_2_S gas generation was higher for *x* = 0.43 and *z* = 0.43, suggesting that higher amount of LiI promoted H_2_S gas generation. As LiI can easily react with moisture, solid electrolytes prepared from high amount of LiI may react with moisture easily. Next, the concentration of H_2_S decreased. This phenomenon may be attributed to the lower speed of H_2_S gas emission than the speed of oxidation of H_2_S gas in air and/or dissolution of H_2_S gas in moisture.^35,36^ The maximum values of H_2_S emitted from *x* = 0 and *z* = 0.37 (200 °C HT) were 1.5 and 2.1 cm^3^ g^−1^, respectively. Calpa *et al.* reported that Li_4_PS_4_I showed higher air stability than Li_3_PS_4_ due to the formation of LiI·H_2_O, which acted as a barrier between PS_4_^3−^ units and H_2_S.^37^ In the present study on Li_4_SnS_4_ with LiI, LiI·3H_2_O (ICSD #759794) was formed after air exposure irrespective of the presence of Li_3_PS_4_ (Fig. S8†). As a result, although Li_3_PS_4_ showed lower air stability than Li_4_SnS_4_,^14^ addition of LiI suppressed the decomposition of Li_3_PS_4_ in LiI–Li_3_PS_4_–Li_4_SnS_4_ type solid electrolytes. Therefore, *z* = 0.37 in *z*LiI·(1 − *z*) (0.6Li_4_SnS_4_·0.4Li_3_PS_4_) heated at 200 °C showed a higher ionic conductivity of 5.5 × 10^−4^ S cm^−1^ at 30 °C and a high air stability similar to Li_4_SnS_4_. Fig. S8† shows that diffraction peaks, attributed to SnS_2_, were observed at 15.0° and 32.2° in the XRD patterns after air exposure, suggesting partial decomposition of solid electrolytes. In this study, solid electrolyte powder was used for air stability measurements. Note that powdered sample was easier to be influenced by moisture than pelletized samples because of the availability of larger surface areas. Further investigation is required to study the decomposition mechanism and make guidelines for suppression of H_2_S gas emission.

## Conclusions

Hexagonal Li_4_SnS_4_-based solid electrolytes with LiI and/or Li_3_PS_4_ were prepared by mechanochemical treatment to enhance the ionic conductivity of hexagonal Li_4_SnS_4_. The sample with *x* = 0.43 in *x*LiI·(1 − *x*)Li_4_SnS_4_ showed higher ionic conductivity of 1.6 × 10^−4^ S cm^−1^ at room temperature than the sample with *x* = 0 (4.5 × 10^−5^ S cm^−1^). We considered that higher ionic conductivities of Li_4_SnS_4_ with LiI resulted from the higher amount of Li carrier due to the increase in the Li/Sn ratio and the expansion of conduction paths of Li^+^ ion due to the introduction of I^−^, which has a large ionic radius. PDF analysis and Raman spectroscopy suggested that I^−^ ions existed between SnS_4_^4−^ units. As-milled samples of Li_4_SnS_4_ with LiI and Li_3_PS_4_ formed the amorphous state. The sample with *z* = 0.37 in *z*LiI·(1 − *z*)(0.4Li_3_PS_4_·0.6Li_4_SnS_4_), after heat-treatment at 200 °C, exhibited ionic conductivity of 5.5 × 10^−4^ S cm^−1^ at 30 °C with an activation energy of 28 kJ mol^−1^. It showed the highest ionic conductivity in hexagonal Li_4_SnS_4_-based solid electrolytes. All-solid-state batteries using NMC composite positive electrodes with *z* = 0.37 (heated at 200 °C) sample showed the highest rate performance compared to the samples where *x* = 0 and *x* = 0.43. Furthermore, the sample with *z* = 0.37 heated at 200 °C showed similar air stability to Li_4_SnS_4_. Formation of LiI·3H_2_O prevents further decomposition of Li_3_PS_4_ in LiI–Li_3_PS_4_–Li_4_SnS_4_ solid electrolytes. We believe that hexagonal Li_4_SnS_4_-based solid electrolytes with additives will keep being developed in the future because of their high ionic conductivity and air stability. Our results provide a new strategy for increasing the ionic conductivity of hexagonal Li_4_SnS_4_ without decreasing the air stability by mechanochemical treatment. Future work in this area will focus on the addition of other components, such as oxide materials, to improve the electrolyte stability toward lithium.

## Author contributions

M. O. developed the idea and conducted experiments. K. K. and H. K. supervised the work. All authors discussed the results and prepared the manuscript. All authors have given approval to the final version of the manuscript.

## Conflicts of interest

There are no conflicts to declare.

## Supplementary Material

RA-011-D1RA06466E-s001

## References

[cit1] Gao Z., Sun H., Fu L., Ye F., Zhang Y., Luo W., Huang Y. (2018). Adv. Mater..

[cit2] Takada K. (2018). J. Power Sources.

[cit3] Kamaya N., Homma K., Yamakawa Y., Hirayama M., Kanno R., Yonemura M., Kamiyama T., Kato Y., Hama S., Kawamoto K., Mitsui A. (2011). Nat. Mater..

[cit4] Kato Y., Hori S., Saito T., Suzuki K., Hirayama M., Mitsui A., Yonemura M., Iba H., Kanno R. (2016). Nat. Energy.

[cit5] Adeli P., Bazak J. D., Park K. H., Kochetkov I., Huq A., Goward G. R., Nazar L. F. (2019). Angew. Chem., Int. Ed..

[cit6] Mizuno F., Hayashi A., Tadanaga K., Tatsumisago M. (2005). Adv. Mater..

[cit7] Seino Y., Ota T., Takada K., Hayashi A., Tatsumisago M. (2014). Energy Environ. Sci..

[cit8] Sakuda A., Hayashi A., Tatsumisago M. (2013). Sci. Rep..

[cit9] Kato A., Nose M., Yamamoto M., Sakuda A., Hayashi A., Tatsumisago M. (2018). J. Ceram. Soc. Jpn..

[cit10] Sahu G., Lin Z., Li J., Liu Z., Dudney N., Liang C. (2014). Energy Environ. Sci..

[cit11] Kaib T., Haddadpour S., Kapitein M., Bron P., Schröder C., Eckert H., Roling B., Dehnen S. (2012). Chem. Mater..

[cit12] Park K. H., Oh D. Y., Choi Y. E., Nam Y. J., Han L., Kim J. Y., Xin H., Lin F., Oh S. M., Jung Y. S. (2016). Adv. Mater..

[cit13] Choi Y. E., Park K. H., Kim D. H., Oh D. Y., Kwak H. R., Lee Y. G., Jung Y. S. (2017). ChemSusChem.

[cit14] Kanazawa K., Yubuchi S., Hotehama C., Otoyama M., Shimono S., Ishibashi H., Kubota Y., Sakuda A., Hayashi A., Tatsumisago M. (2018). Inorg. Chem..

[cit15] Kimura T., Kato A., Hotehama C., Sakuda A., Hayashi A., Tatsumisago M. (2019). Solid State Ionics.

[cit16] Kwak H., Park K. H., Han D., Nam K. W., Kim H., Jung Y. S. (2020). J. Power Sources.

[cit17] Zhang Z., Zhang J., Sun Y., Jia H., Peng L., Zhang Y., Xie J. (2020). J. Energy Chem..

[cit18] Matsuda R., Kokubo T., Phuc N. H. H., Muto H., Matsuda A. (2020). Solid State Ionics.

[cit19] Leube B. T., Inglis K. K., Carrington E. J., Sharp P. M., Shin J. F., Neale A. R., Manning T. D., Pitcher M. J., Hardwick L. J., Dyer M. S., Blanc F., Claridge J. B., Rosseinsky M. J. (2018). Chem. Mater..

[cit20] Minafra N., Culver S. P., Li C., Senyshyn A., Zeier W. G. (2019). Chem. Mater..

[cit21] Mercier R., Malugani J.-P., Fahys B., Robert G. (1981). Solid State Ionics.

[cit22] Ujiie S., Hayashi A., Tatsumisago M. (2012). Solid State Ionics.

[cit23] Rangasamy E., Liu Z., Gobet M., Pilar K., Sahu G., Zhou W., Wu H., Greenbaum S., Liang C. (2015). J. Am. Chem. Soc..

[cit24] Suyama M., Kato A., Sakuda A., Hayashi A., Tatsumisago M. (2018). Electrochim. Acta.

[cit25] Kato A., Yamamoto M., Sakuda A., Hayashi A., Tatsumisago M. (2018). ACS Appl. Energy Mater..

[cit26] Muramatsu H., Hayashi A., Ohtomo T., Hama S., Tatsumisago M. (2011). Solid State Ionics.

[cit27] Tatsumisago M., Hama S., Hayashi A., Morimoto H., Minami T. (2002). Solid State Ionics.

[cit28] Sakuda A., Takeuchi T., Kobayashi H. (2016). Solid State Ionics.

[cit29] Izumi F., Momma K. (2007). Solid State Phenom..

[cit30] Ohta N., Takada K., Sakaguchi I., Zhang L., Ma R., Fukuda K., Osada M., Sasaki T. (2007). Electrochem. Commun..

[cit31] Pohl S., Schiwy W., Weinstock N., Krebs B. (1973). Z. Naturforsch., B: Anorg. Chem., Org. Chem..

[cit32] Takahashi M., Watanabe T., Yamamoto K., Ohara K., Sakuda A., Kimura T., Yang S., Nakanishi K., Uchiyama T., Kimura M., Hayashi A., Tatsumisago M., Uchimoto Y. (2021). Chem. Mater..

[cit33] Otoyama M., Kuratani K., Kobayashi H. (2021). Ceram. Int..

[cit34] Otoyama M., Sakuda A., Tatsumisago M., Hayashi A. (2020). ACS Appl. Mater. Interfaces.

[cit35] Hales J. M., Wilkes J. O., York J. L. (1974). Tellus.

[cit36] Balls P. W., Liss P. S. (1983). Atmos. Environ..

[cit37] Calpa M., Rosero-Navarro N. C., Miura A., Jalem R., Tateyama Y., Tadanaga K. (2021). Appl. Mater. Today.

