# Supplementary material for: Mechanochemical synthesis of air-stable hexagonal Li4SnS4-based solid electrolytes containing LiI and Li3PS4
Source: RSC Adv. 2021 Dec 3;11(61):38880–8. doi: 10.1039/d1ra06466e (PMC9044197; doi:10.1039/d1ra06466e)
Supplement: RA-011-D1RA06466E-s001 [file RA-011-D1RA06466E-s001.pdf]

Electronic Supplementary Material (ESI) for RSC Advances.
This journal is © The Royal Society of Chemistry 2021

Supplementary information

Mechanochemical synthesis of air-stable hexagonal Li_4SnS_4-based solid electrolytes containing LiI and Li_3PS_4

Misae Otoyama,* Kentaro Kuratani, and Hironori Kobayashi

Research Institute of Electrochemical Energy, Department of Energy and Environment, National Institute of Advanced Industrial Science and Technology (AIST), 1-8-31, Midorigaoka, Ikeda, Osaka 563-8577, Japan

Research Institute of Electrochemical Energy, Department of Energy and Environment, National Institute of Advanced Industrial Science and Technology (AIST),
1-8-31, Midorigaoka, Ikeda, Osaka 563-8577, Japan
Tel.: +81-72-751-7932; Fax.: +81-72-751-9609
*Correspondence to: misae-otoyama@aist.go.jp

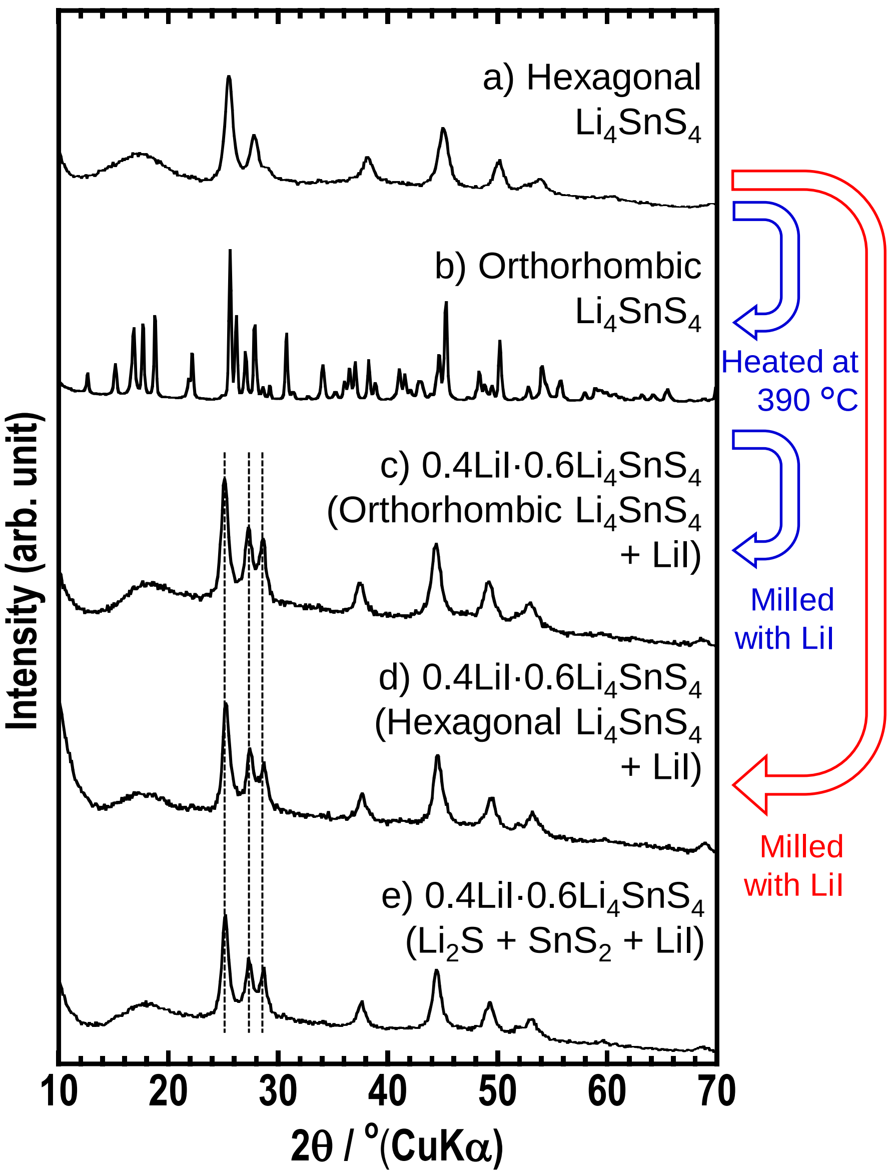

Fig. S1. XRD patterns of a) hexagonal Li_4SnS_4, b) orthorhombic Li_4SnS_4 prepared by heat treatment of a) hexagonal Li_4SnS_4 at 390 °C, c) $0.4LiI{\cdot}0.6Li_4SnS_4$ prepared by ball milling of b) orthorhombic Li_4SnS_4 and LiI, d) $0.4LiI{\cdot}0.6Li_4SnS_4$ prepared by ball milling of a) hexagonal Li_4SnS_4 and LiI, and e) $0.4LiI{\cdot}0.6Li_4SnS_4$ prepared by ball milling of Li_2S, SnS_2 and LiI.

Table S1 Ionic conductivities of xLiI·(1−x)Li_4SnS_4 at 25 °C and 60 °C.

xLiI·(1−x)Li_4SnS_4	$\sigma_{25\ °C}$ /S cm^{-1}	$\sigma_{60\ °C}$ /S cm^{-1}
$x = 0$	4.5×10^{-5}	1.4×10^{-4}
$x = 0.40$	1.1×10^{-4}	4.0×10^{-4}
$x = 0.41$	1.2×10^{-4}	3.9×10^{-4}
$x = 0.42$	1.5×10^{-4}	4.2×10^{-4}
$x = 0.43$	1.6×10^{-4}	4.9×10^{-4}
$x = 0.44$	1.4×10^{-4}	4.4×10^{-4}
$x = 0.45$	1.4×10^{-4}	4.6×10^{-4}
$x = 0.50$	7.7×10^{-5}	4.2×10^{-4}

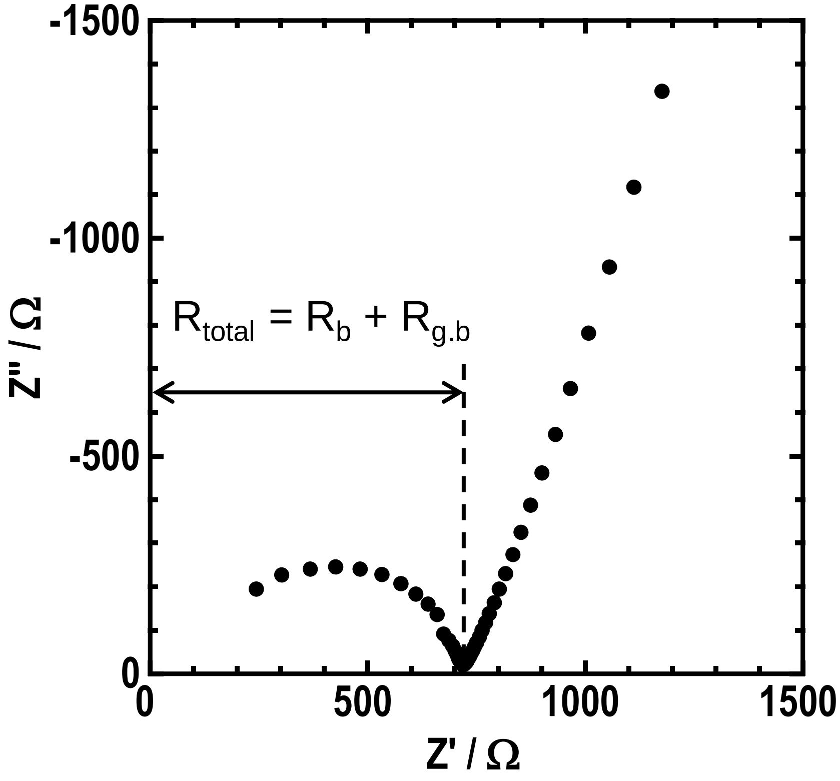

Fig. S2. Nyquist plots of $0.43Li_2S\cdot 0.57Li_4SnS_4$ at 25 °C. The ionic conductivity was determined from the total resistance ($R_{total} = R_b + R_{g.b.}$).

Preparation and characterization of solid electrolytes on Li_2S–SnS_2–LiI system with various Li_2S/SnS_2 ratios of 67/33, 70/30 and 75/25.

The Li_2S–SnS_2 solid electrolytes were prepared with various Li_2S/SnS_2 ratios of 67/33, 70/30 and 80/20. Note that the 67/33 sample is the same as Li_4SnS_4. Fig. S3a shows XRD patterns of these samples. The 67/33 and 70/30 samples exhibit the diffraction peaks of hexagonal Li_4SnS_4 mainly. However, the diffraction peaks of Li_2S were observed in the 70/30 and 80/20 samples, indicating that all Li_2S was not used to reaction in the case of the ratio beyond 67/33. The ionic conductivities of these samples were measured by A.C. impedance (Table S2). The ionic conductivities of the 70/30 and 80/20 samples are lower than that of the 67/33 sample, suggesting that Li_2S decreased their ionic conductivities. Therefore, increasing the amount of Li_2S in the Li_2S–SnS_2 system is not an effective way to increase Li carrier and ionic conductivity.

Fig. S3b displays the XRD patterns of LiI-added $67Li_2S\cdot33SnS_2$, $70Li_2S\cdot30SnS_2$, and $80Li_2S\cdot20SnS_2$. The $0.4LiI\cdot0.6(80Li_2S\cdot20SnS_2)$ still have the diffraction peaks of Li_2S while $0.4LiI\cdot0.6(70Li_2S\cdot30SnS_2)$ has no peaks of Li_2S, indicating that Li_2S remained though LiI was added in the case of $80Li_2S\cdot20SnS_2$. Addition of LiI also increases ionic conductivities of 70/30 and 80/20 samples, while the 67/33 sample with LiI shows the highest ionic conductivity. (Table S2)

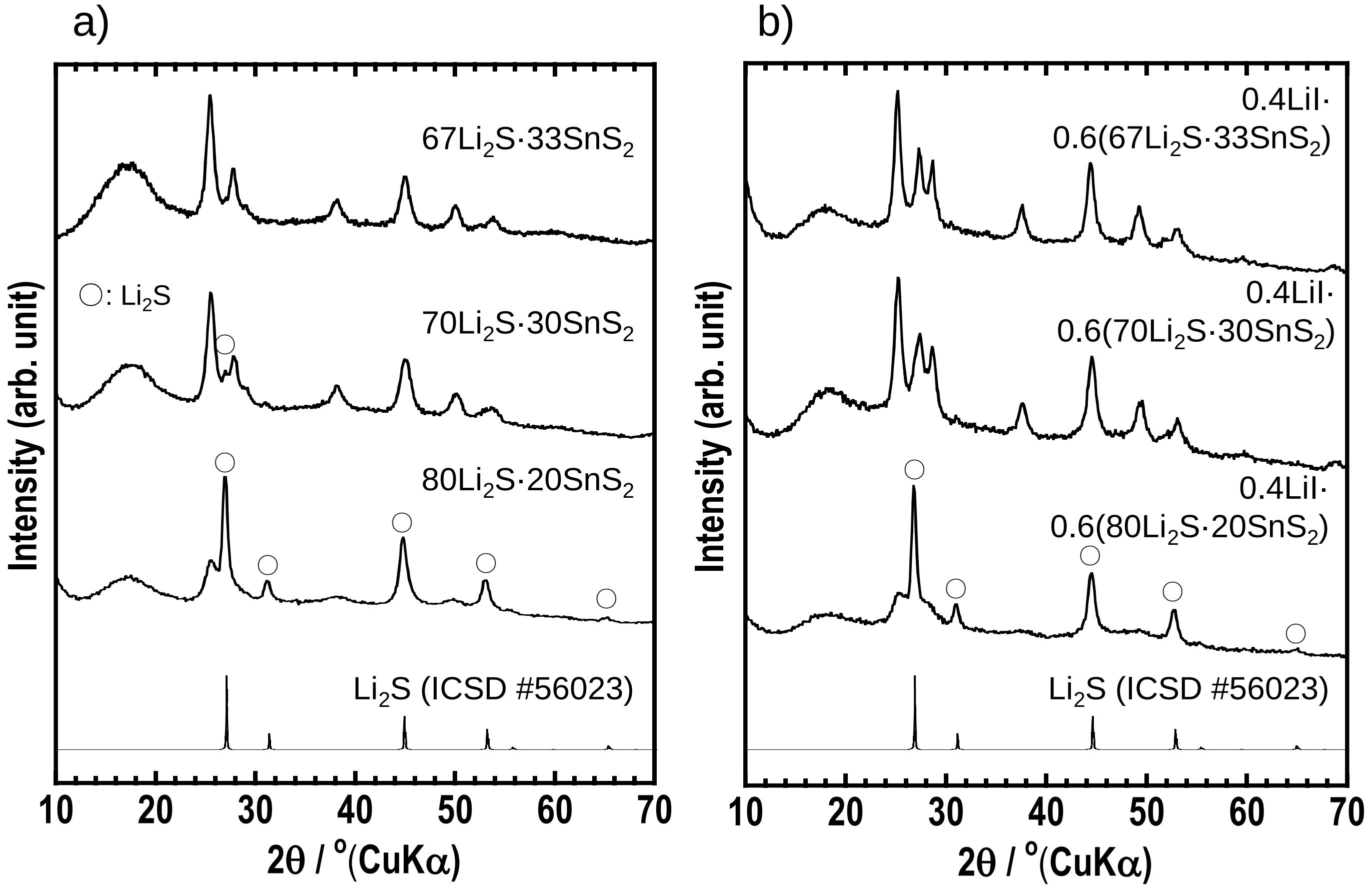

Fig. S3. XRD patterns of a) $67Li_2S\cdot33SnS_2$, $70Li_2S\cdot30SnS_2$ and $80Li_2S\cdot20SnS_2$ and b) LiI-added $67Li_2S\cdot33SnS_2$, $70Li_2S\cdot30SnS_2$ and $80Li_2S\cdot20SnS_2$.

Table S2 Ionic conductivities at room temperature (25 °C) of $67Li_2S\cdot33SnS_2$, $70Li_2S\cdot30SnS_2$ and $80Li_2S\cdot20SnS_2$ solid electrolytes with and without 0.4 mol LiI.

	without LiI (S cm^{-1})	with LiI (S cm^{-1})
$67Li_2S\cdot33SnS_2$	4.5×10^{-5}	1.1×10^{-4}
$70Li_2S\cdot30SnS_2$	4.2×10^{-5}	7.3×10^{-5}
$80Li_2S\cdot20SnS_2$	1.9×10^{-5}	5.8×10^{-5}

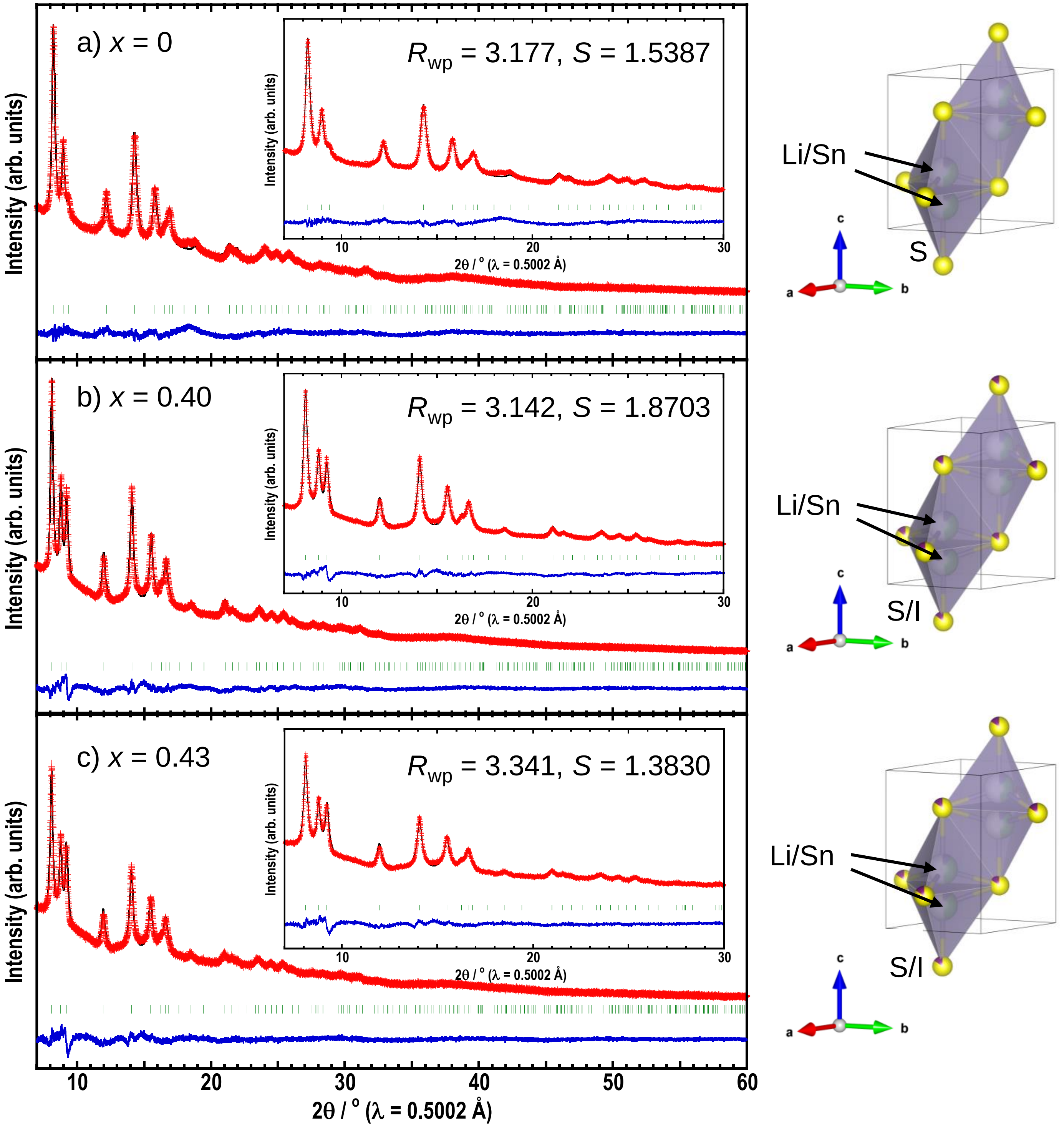

Fig. S4. Synchrotron XRD patterns and structures of a) x = 0, b) x = 0.40 and c) x = 0.43 in xLiI·(1−x)Li_4SnS_4. The red crosses, black continuous line, and bottom blue line represents the observed, calculated, and difference patterns, respectively. Bottom vertical green bars are the Bragg positions.

Table S3 Crystallographic data of Li_4SnS_4.

Space group: $P6_3/mmc$ (No.194), $a = b$ = 4.0174(6) Å, c = 6.3935(9) Å, V = 89.36(2) $Å^3$

atom	site	g	x	y	z	B ($Å^2$)
S	2*c*	1	1/3	2/3	1/4	2.58(6)
Sn	4*f*	0.125	2/3	1/3	0.14372(17)	=S
Li	4*f*	0.375	2/3	1/3	=Sn	=S

Reliability parameters: R_{wp} = 3.177, S = 1.5387

Table S4 Crystallographic data of $0.4LiI·0.6Li_4SnS_4$.

Space group: $P6_3/mmc$ (No.194), $a = b$ = 4.07114(18) Å, c = 6.5076(4) Å, V = 93.409(8) $Å^3$

atom	site	g	x	y	z	B ($Å^2$)
S	2*c*	0.857	1/3	2/3	1/4	1.61(3)
I	2*c*	0.143	1/3	2/3	1/4	=S
Sn	4*f*	0.107	2/3	1/3	0.13750(19)	=S
Li	4*f*	0.375	2/3	1/3	=Sn	=S

Reliability parameters: R_{wp} = 3.142, S = 1.8703

Table S5 Crystallographic data of $0.43LiI·0.57Li_4SnS_4$.

Space group: $P6_3/mmc$ (No.194), $a = b$ = 4.0822(7) Å, c = 6.5311(9) Å, V = 94.26(2) $Å^3$

atom	site	g	x	y	z	B ($Å^2$)
S	2*c*	0.841	1/3	2/3	1/4	1.44(7)
I	2*c*	0.159	1/3	2/3	1/4	=S
Sn	4*f*	0.105	2/3	1/3	0.1363(3)	=S
Li	4*f*	0.375	2/3	1/3	=Sn	=S

Reliability parameters: R_{wp} = 3.341, S = 1.3830

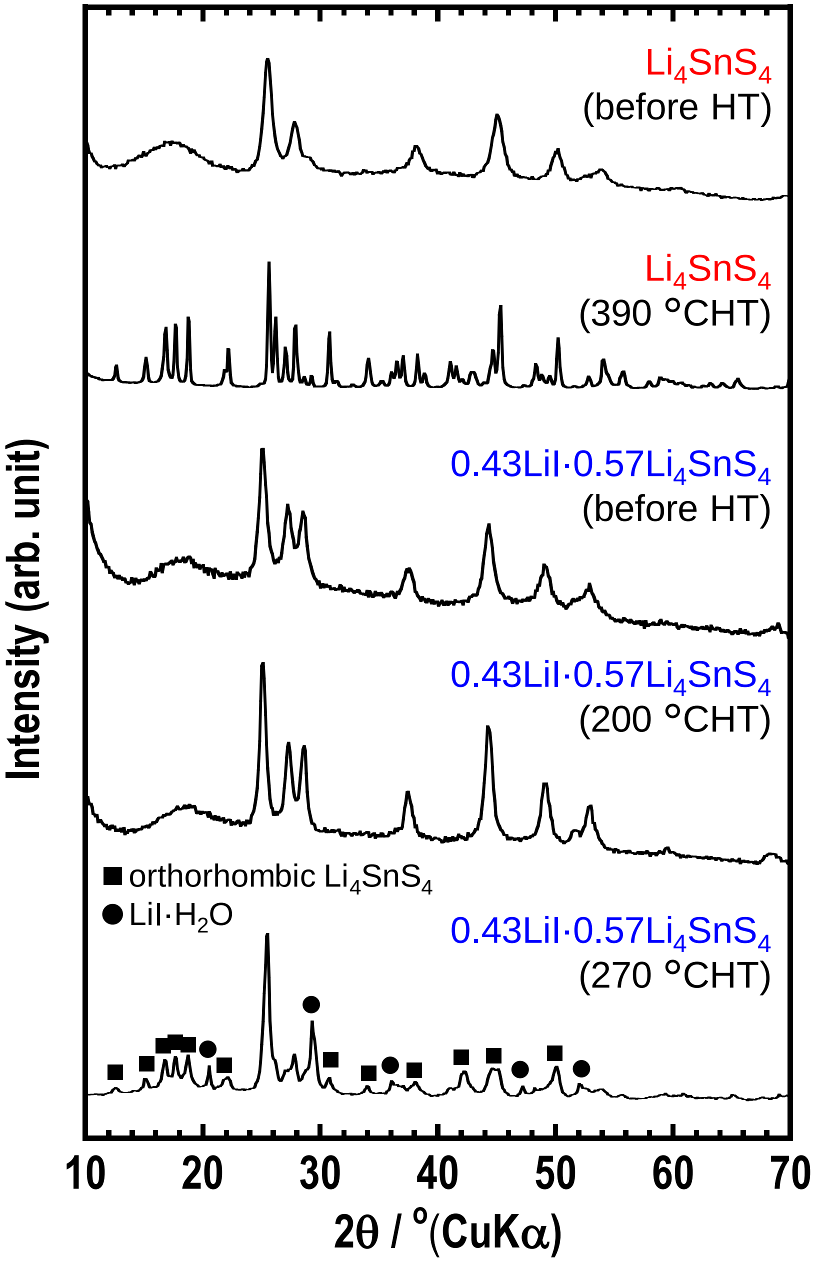

Fig. S5. XRD patterns of x = 0 and x = 0.43 in xLiI·(1−x)Li_4SnS_4 before and after the heat treatment (HT). The x = 0 and x = 0.43 were heated at 390 °C and 200 °C/270 °C, respectively.

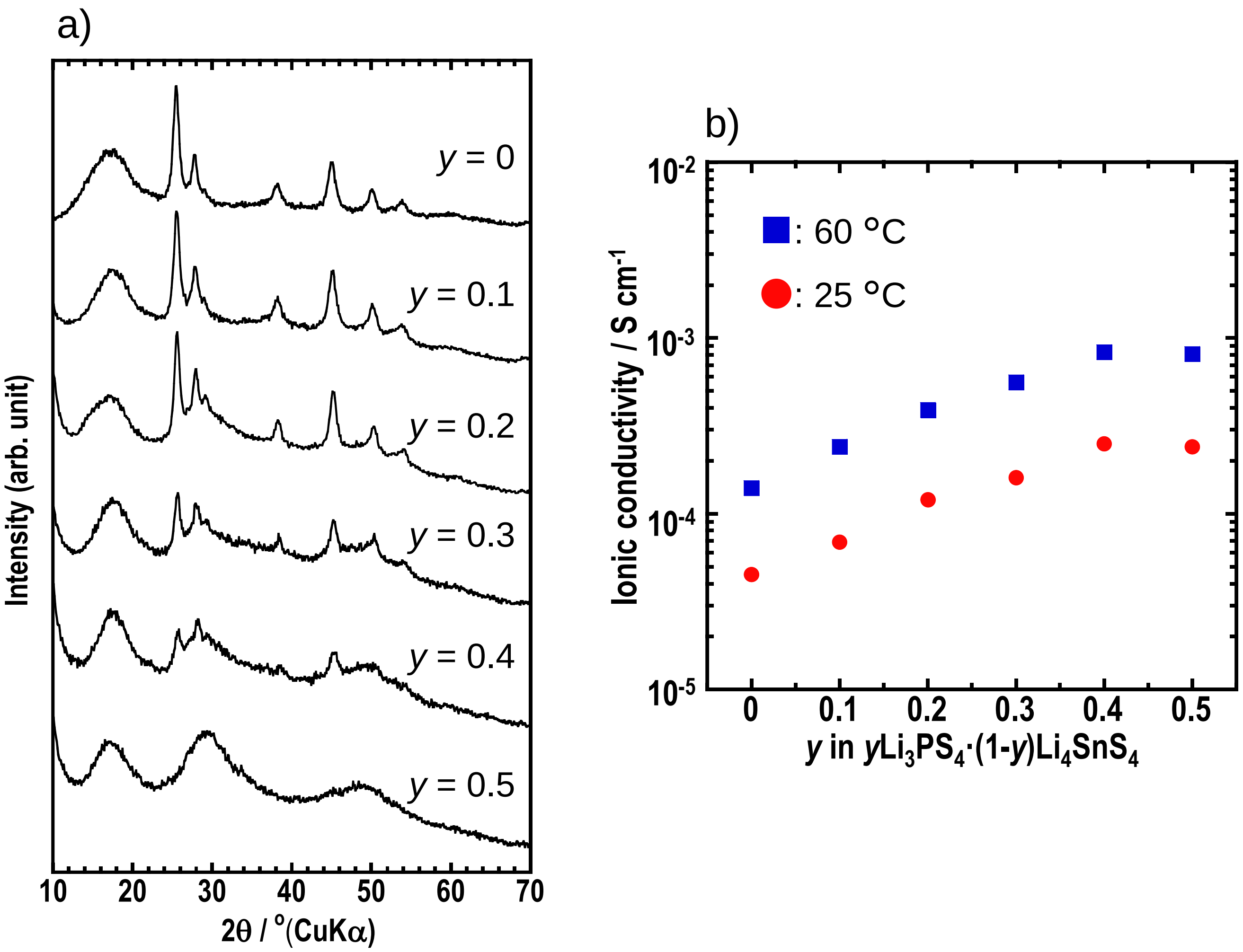

Fig. S6. a) XRD patterns and b) ionic conductivities at 25 °C and 60 °C of y = 0–0.5 in $y\mathrm{Li_3PS_4}\cdot(1-y)\mathrm{Li_4SnS_4}$.

Table S6 The activation energies and ionic conductivities at 30 °C of x = 0 and x = 0.43 in xLiI·(1−x)Li_4SnS_4, z = 0.37, z = 0.37 (200 °C HT) and z = 0.43 in zLiI·(1−z)(0.4Li_3PS_4·0.6Li_4SnS_4), and 0.4LiI·0.6Li_4SnS_4 glass prepared liquid-phase synthesis.

	E_a / kJ mol^{-1}	$\sigma_{30\ °C}$ /S cm^{-1}
Li_4SnS_4	37	3.7×10^{-5}
0.43LiI·0.57Li_4SnS_4	39	1.0×10^{-4}
0.37LiI·0.63(0.4Li_3PS_4·0.6Li_4SnS_4)	33	4.4×10^{-4}
0.37LiI·0.63(0.4Li_3PS_4·0.6Li_4SnS_4) 200 °C HT	28	5.5×10^{-4}
0.43LiI·0.57(0.4Li_3PS_4·0.6Li_4SnS_4)	32	5.5×10^{-4}
0.4LiI·0.6Li_4SnS_4 glass (Liquid-phase synthesis)*	41.6	4.1×10^{-4}

*data from the reference [12] "K. H. Park, D. Y. Oh, Y. E. Choi, Y. J. Nam, L. Han, J. Y. Kim, H. Xin, F. Lin, S. M. Oh and Y. S. Jung, *Adv. Mater.*, 2016, **28**, 1874–1883."

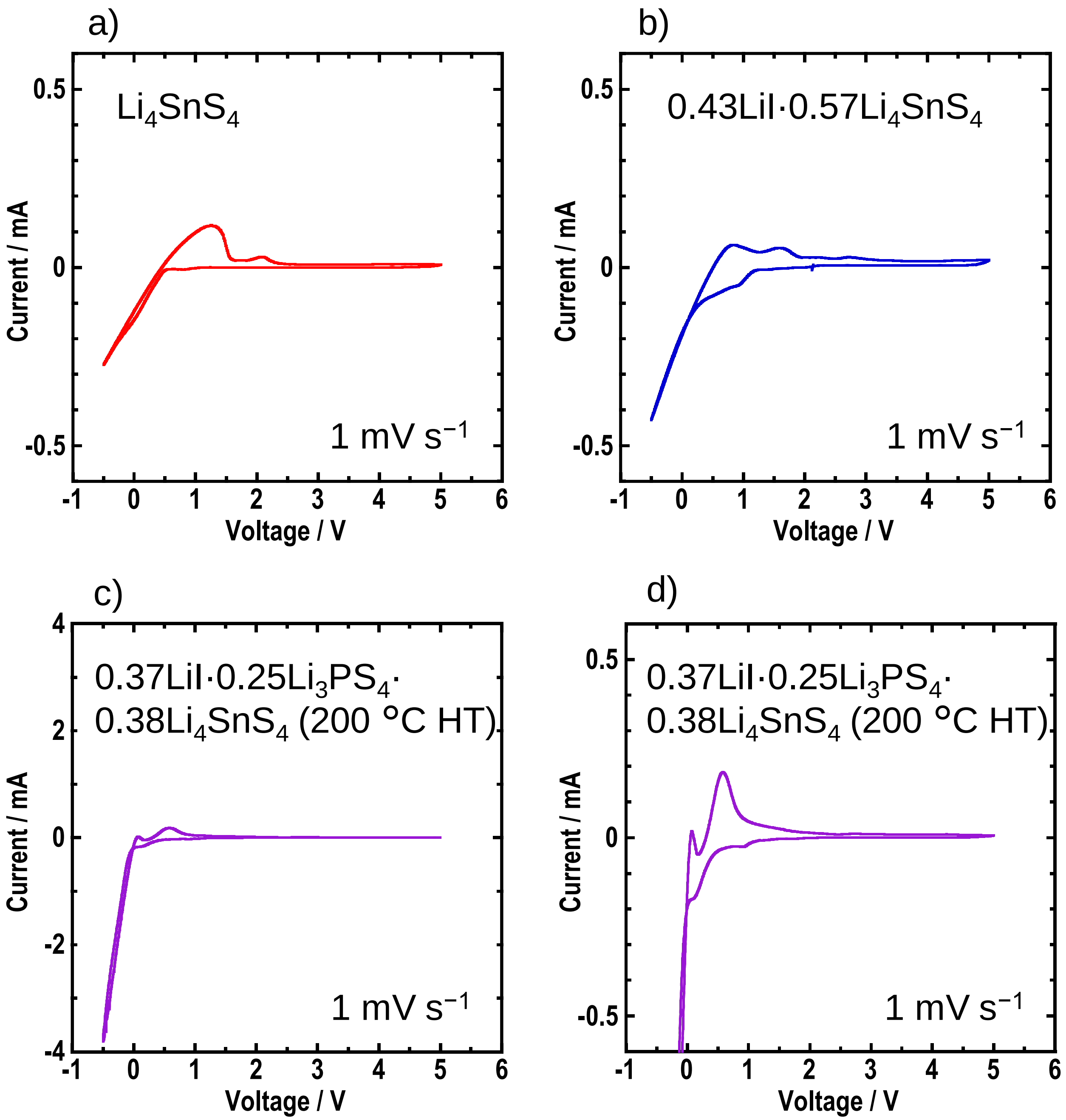

Fig. S7. Cyclic voltammograms of the Li/solid electrolytes/SUS asymmetric cells using a) Li_4SnS_4, b) $0.43LiI{\cdot}0.57Li_4SnS_4$, and c) $0.37LiI{\cdot}0.25Li_3PS_4{\cdot}0.38Li_4SnS_4$ heated at 200 °C as solid electrolytes from −0.5 to 5 V. The scan rate was 1 mV s^{-1}. d) An enlarged view of Fig. S7c with a current range of −0.6 and 0.6 mA.

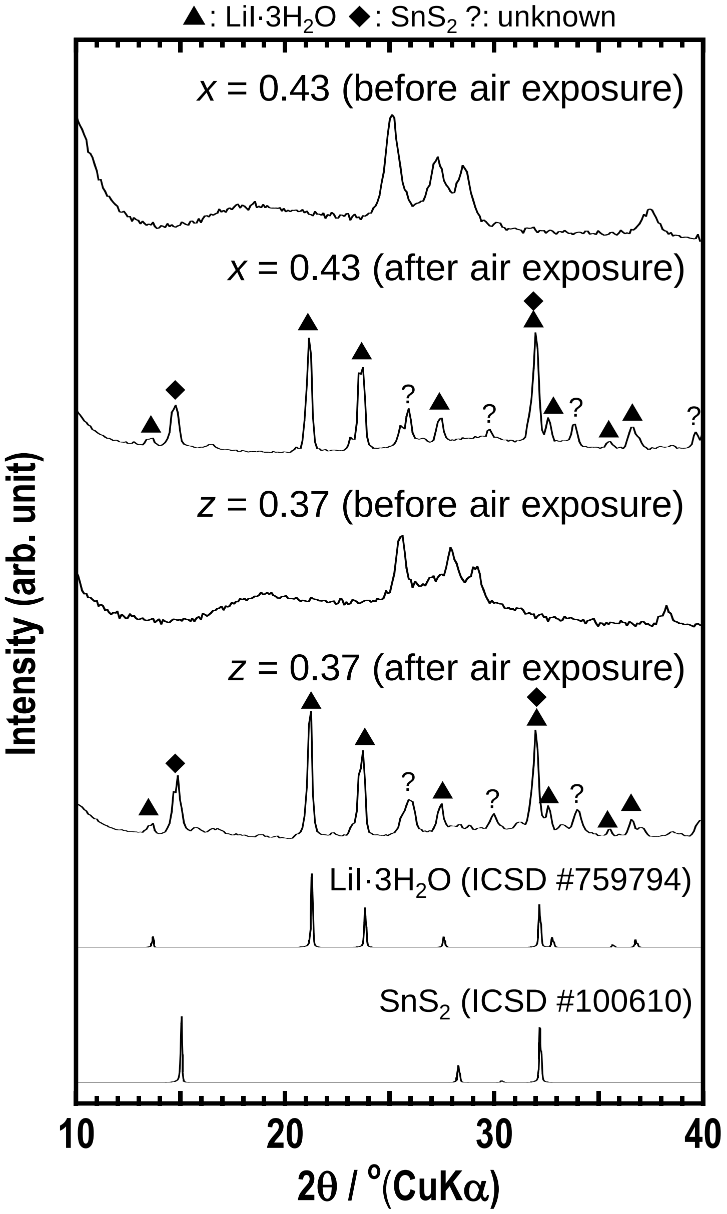

Fig. S8. XRD patterns of x = 0.43 in xLiI·(1−x)Li_4SnS_4 and z = 0.37 in zLiI·(1−z)(0.4Li_3PS_4·0.6Li_4SnS_4) before and after the air exposure.
